# Evaluation of cannabinoid receptors type 1–2 in periodontitis patients

**DOI:** 10.1002/cre2.608

**Published:** 2022-06-19

**Authors:** Atefe Ataei, S. A. Rahim Rezaee, Amir Moeintaghavi, Habibollah Ghanbari, Majid Azizi

**Affiliations:** ^1^ Department of Periodontics, School of Dentistry Birjand University of Medical Sciences Birjand Iran; ^2^ Immunology Research Center, Inflammation and Inflammatory Diseases Division, Faculty of Medicine Mashhad University of Medical Sciences Mashhad Iran; ^3^ Department of Periodontology, School of Dentistry Mashhad University of Medical Sciences Mashhad Iran; ^4^ Department of Orthodontics, School of Dentistry Birjand University of Medical Sciences Birjand Iran

**Keywords:** CB1, CB2, endocannabinoids, periodontitis

## Abstract

**Background:**

As effective immune modulators, Endocannabinoids may suppress the inflammatory responses in periodontitis. This study assessed the expression of cannabinoid receptors in gingiva and the impact on periodontitis.

**Methods:**

A cross‐sectional study on 20 patients with more than stage II and Grade A periodontitis and a control group consisting of 19 healthy individuals was performed. The gingival biopsies were assessed for the expression of *CB1* and *CB2* using the quantitative reverse transcription polymerase chain reaction, TaqMan method.

**Results:**

The study sample consisted of 39 subjects, 31 females (79.5%) and 8 males (20.5%), including 20 periodontitis subjects (80% female and 20% male), and control groups (78.9% female and 21.1% male). The mean ages of cases and controls were 33.3 ± 4.7 and 35.7 ± 5.1 years, respectively. The gene expression of CB2 in periodontitis was 27.62 ± 7.96 and in healthy subjects was 78.15 ± 23.07. The CB2 was significantly lower than the control group (*p* = .008). In comparison, the gene expression index of CB1 in the periodontal group (9.42 ± 3.03) was higher than the control group (6.62 ± 1.13) but did not meet a significant value (*p* = .671).

**Conclusion:**

The lower expression of CB2 receptors in the periodontitis group may be due to the reduced protective effect of anti‐inflammatory agents. These elements include cannabinoids and the imbalance leading to the predominance of pro‐inflammatory effects. Therefore, the local effects of cannabinoids as an immunomodulator could be useful for oral inflammatory diseases such as periodontitis.

## 1 INTRODUCTION

Periodontitis, oro‐dental infection, and inflammatory disease is highly prevalent worldwide. This gingival microbial infection affects around 10–15% of the population (Al‐Mutairi et al., 2022; Chapple et al., 2015). The genetic, epigenetics, and environmental factors play significant roles in the susceptibility to this disease, such as the nature of immune responses, smoking, lack of proper oral health, and *Porphyromonas gingivalis* infection (Kinane & Lappin, 2002; Loos et al., 2020). Periodontitis is more frequent and is known as a multifactorial nonreversible inflammatory disease (Lang et al., 2009). Some types of the disease are progressive and may be exacerbated by systemic or environmental factors that alter the host's response to plaque accumulation, such as immunodeficiency, diabetes, smoking, and stress (Page & Eke, 2007).

In almost all types, periodontal pathogens and the host defense system play key roles in the onset and progression of these diseases. The periodontal disease as destructive lesions in gingiva and tooth‐supporting tissues, such as alveolar bone and periodontal ligament, might be an imbalance of the microbial‐host interactions at the site of infection. The inflammatory reactions may be limited to subjects prone to the destructive outcome and promoted to periodontitis (Highfield, 2009). The disastrous events start with a microbial infection, overgrowth, and accumulation of dental plaque. These events are followed by host inflammatory reactions, which recruit leukocytes and induce cytokines, eicosanoids, matrix metalloproteinase (MMP) production, and, finally, injury to the tissue (AlMoharib et al., 2014). Therefore, from these pro‐inflammatory cytokines, interleukins‐1 (IL‐1), IL‐6, IL‐8, IL‐12, interferon‐γ, IL‐18, and tumor necrosis factor‐α (TNF‐α), substantial inflammatory factors including IL‐1β, TNF‐α, IL‐18, collagenase, and MMP‐8, and IL‐9 in saliva can be detected (Äyräväinen et al., 2018; Kaufman & Lamster, 2000; Miller et al., 2006; Moeintaghavi et al., 2017).

One of the findings in the field of immune‐regulatory factors is identifying the endocannabinoid (EC) system, which could modulate the immune responses, particularly T‐ and B‐lymphocytes migration, proliferation, and proliferation cytokine production (Braile et al., 2021; Cencioni et al., 2010; Klein et al., 2003). The previously studied results on the EC system brought them to the attention of researchers as potential medications for introducing a novel target for many hypersensitivity diseases (Croxford & Yamamura, 2005; Tanasescu & Constantinescu, 2010) such as multiple sclerosis (MS; Sativex, an oromucosal spray; Giacoppo et al., 2017).

The major cannabinoid receptors, CB1 and CB2, are G‐binding receptors inducing selective transcription factors, depending on the cell and the receptor types. Moreover, they might have modulatory effects on the immune system and control various activities in the brain such as learning and memory, mood, pain perception, and healing events in neural damages (Reggio, 2010). However, the CB1 and CB2 activities have been introduced in many immune tissues and cells (Bab et al., 2008; Croxford & Yamamura, 2005).

Gingival fibroblasts express both CB1 and CB2 receptors involved in tissue and wound repair and were upregulated under pathological conditions in periodontal inflammation (Nakajima et al., 2006). Furthermore, Nakajima et al. (2006) showed that Anandamide (AEA) inhibited the main inflammation pathway triggered by the nuclear factor‐κB (NF‐κB) pathway, leading to regulating hyperinflammatory reactions in periodontitis. ECs, as effective immune modulators, may suppress the inflammatory responses in periodontitis. On the other hand, in ECs, decreased number of inflammatory epithelial responses to bacterial stimuli may lead to immune escape colonization and resistance to bacteria should be aware of evading infection. However, such advances in ECs and the introduction of the recent cannabinoid medications are a new pathway for preventing destructive inflammatory reactions such as periodontitis. Besides, for a better understanding of the immunomodulatory mechanisms of cannabinoid receptors, their distribution and impact must be considered.

Therefore, this study assessed the expressions of cannabinoid receptors in healthy gingiva and periodontitis lesions.

## 2 MATERIAL AND METHODS

### 2.1 Study population

A cross‐sectional study was arranged from April 2017 to November 2017 at the Dental Faculty of Mashhad University of Medical Sciences (MUMS). The MUMS ethics committee approved the study (IR.mums.sd.REC.1394.296). Gingiva tissue samples during surgery with a size of 3–5mm in length (anterior–posterior direction) and 1–3 mm in width were collected from 20 subjects with more than Stage II and Grade A periodontitis (16 women and 4 men, mean age 33.3 ± 4.7 years), and from 19 healthy individuals in terms of gingival and periodontal conditions, who were treated for crown lengthening (11 women and 8 men, mean age 35.7 ± 5.1 years). Several exclusion criteria were considered such as systemic disease affecting periodontal conditions or the need for antibiotic prophylaxis, having before surgical treatments, systemic or local use of antimicrobials or anti‐inflammatory drugs in the last 6 months, pregnancy, lactation, or the use of contraceptives in the women studied, the existence of lesions and endodontic changes in the studied teeth, and smoking. According to the World Health Organization recommendations, periodontal parameters were documented (World Health Organization, 1997).

### 2.2 RNA extraction, complementary DNA synthesis

Total RNA from gingival tissue samples was isolated using TriPure TM Reagent (Roche Diagnostics, Lewes, UK). The RNA concentration was determined by NanoPhotometry (Implant, Germany) at 260 and 280 nm. The single‐stranded cDNA was synthesized by RevertAid™ H Minus‐Mulv reverse transcriptase (Fermentas Co, Germany) using 0.1 µg Oligo (dT)18‐primer in a Thermal cycler (Mastercycler, Eppendorf, Westbury, NY).

### 2.3 Real‐time quantitative polymerase chain reaction (PCR)

The real‐time TaqMan PCR relative method with a Rotor‐Gene Q Machine (Qiagen, Hilden, Germany) and the Universal Master Mix (Takara, Otsu Shiga, Japan) quantify the CB1 and CB2 expression in the 39 cDNAs. A housekeeping gene, β2‐microglobulin, was simultaneously processed for each sample. A two‐standard curve technique was employed to determine the amplification efficiencies.

Primers and probes were designed using Beacon Designer software version 7 (Beacon Business Systems, Australia) using TaqMan and SYBR Green assays for *CB1* and *CB2* gene expression. Table 1 lists the respective forward, reverse, and probe primer.

**Table 1:** The oligonucleotide and probe sequences

Targeted gene	Sequence (5′ → 3′)	Purpose	Product size (bp)
*CB1*	Forward	GTGTTCCACCGCAAAGATAGC	130 bp
	Reverse	GGGGCCTGTGAATGGATATGT	
	Probe	CCTCCGTGGGCAGCCTGTTCCTCA‐BHQ‐1	
*CB2*	Forward	GGGTGACAGAGATAGCCAATGG	204 bp
	Reverse	TGAACAGGTATGAGGGCTTCC	
*β2m*	Forward	TTGTCTTTCAGCAAGGACTGG	127 bp
	Reverse	CCACTTAACTATCTTGGGCTGTG	
	Probe	TCACATGGTTCACACGGCAGGCAT‐BHQ‐1	

Abbreviation: bp, base pairs.

Gene expression was measured by relative quantification, which compares each transcript's threshold cycle (Ct) normalized to the internal control.

### 2.4 Statistical analysis

Statistical analyses were performed with SPSS software (SPSS, Inc., Chicago, IL, USA). Descriptive data were expressed as means ± SD. Differences between two groups were calculated using the Mann–Whitney *U* test for nonparametric data according to the normality Kolmogorov–Smirnov test to evaluate differences in continuous variables between groups. Spearman rank correlation test was performed to assess the association between age and CB1 and CB2 expression; *p*  <  .05 was taken as significant.

## 3 RESULTS

### 3.1 Study population and demographic data

The study sample consisted of 39 subjects, 31 females (79.5%) and 8 males (20.5%), including 20 periodontal cases (80% female and 20% male) and control groups (78.9% female and 21.1% male). The mean ages of cases and controls were 33.3 ± 4.7 and 35.7 ± 5.1 years, respectively. No significant difference existed between gender and age.

### 3.2 Gene expression findings

Due to the lack of normal distribution of *CB2* gene expression, the Mann–Whitney *U* test assessed the difference between *CB2* expressions in the two groups. The gene expression of *CB2* in periodontitis was 27.62 ± 7.96 and in healthy subjects 78.15 ± 23.07. The *CB2* expression was strongly downregulated in periodontitis subjects compared with the controls (*p* = .008).

The gene expression index of CB1 in the periodontal group (9.42 ± 3.03) was higher than the control group (6.62 ± 1.13) but did not meet a significant value (Table 2).

**Table 2:** Gene expression findings

Variable	Case group(20)	Control group	*p*
CB1	9.42 ± 3.03	6.62 ± 1.13	.671^a^
CB2	27.62 ± 7.96	78.15 ± 23.07	.008^a^

^a^ Mann–Withney test.

### 3.3 Correlations

As Table 3 shows, in both periodontitis and control groups, age has a nonsignificant negative correlation with CB1 and CB2; in other words, CB1 and CB2 values decrease with increasing age and they move in opposite directions. Furthermore, there was no significant correlation between CB1 and CB2 expressions in the studied groups.

**Table 3:** Age correlation with CB1 and CB2

Variable	Case group(20)	Control group
CB1	*R* = −0.183, *p* = .416	*R* = −0.195, *p* = .452
CB2	*R* = −0.194, *p* = .387	R = −0.098, *p* = .707

## 4 DISCUSSION

In the present study, the *CB1* and *CB2* gene expression in periodontitis showed a significant presence of CB2 in the gingival tissues, but not CB1. Indeed, the CB1 expression difference in the inflammation site between the control and experimental groups was not significant. CB2 expression was statistically significantly lower in the periodontitis group than in the control group (*p* = .008). As CB1 is mainly expressed in the cells and nerve tissues, it does not seem that, even in theory, it has a pivotal role in the pathogenesis of inflammatory diseases such as periodontitis. Therefore, most studies on cannabinoid receptors in other inflammatory conditions such as rheumatoid arthritis were limited to CB2 receptors and their agonists (Barrie et al., 2017).

In this study, CB2 receptor has been significantly lower than the healthy group, contrary to the study results by Milani et al. (2012) in which the CB2 receptor was significantly higher in the periodontitis group. This difference could be due to the lower sample size (four patients with chronic periodontitis and six healthy individuals) and the techniques.

Nakajima et al. (2006) showed the presence of CB1 and CB2 receptors after bacterial stimulation; therefore, cannabinoid systems may play anti‐inflammatory effects. Furthermore, the AEA effect on the PDL fibroblast cells showed a pro‐inflammatory effect and increased IL‐6 expression. The addition of *P. gingivalis* lipopolysaccharide to the culture medium stimulated AEA to stimulate anti‐inflammatory responses instead of pro‐inflammatory activity (Nakajima et al., 2006).

The study that examined the effect of periodontitis treatment on the activity of the EC system is the study of Kozono et al. (2010), which showed an increase in the AEA after periodontal surgery in patients with periodontitis.

Anti‐inflammatory actions of cannabinoids may be through CB2 surface receptors, because drug inhibition of CB2 receptors does not lead to the reversal of the innate immune response to bacterial stimuli in epithelial cells. One study found that specific inhibitors, such as AM630, and small interfering RNA, an inhibitor that silences the *CB2* gene, could be used to determine the importance of CB2 in inhibiting the innate response to plaque pathogens and gingival epithelial cells (Niyogi, 2016).

The effect of a cellular receptor on inflammatory signaling pathways depends on the presence of ligands affecting it. It is certainly not possible to conclude from a recipient's statement about its role in the disease process without examining the number of its agonists, which is one of the significant limitations of our study and the study of Milani et al. (2012) However, in the study of Nakajima et al. (2006), CB2 receptor agonists were shown in the gingival crevice fluid of patients with periodontitis.

One of the essential points in investigating the role of a receptor–ligand system in periodontal pathogenesis is its effect on key transcription factors involved in expressing inflammatory cytokine genes. As in the pathogenesis of the periodontal disease, the expression of cytokines, especially IL‐1β and TNF‐α, is initiated following the stimulation of bacterial lipopolysaccharide by NF‐κB, the role of a cellular pathway should include in vitro studies showing the effect of this molecular pathway on NF‐κB expression (Nichols et al., 2001). Nakajima et al. (2006) showed AEA's inhibitory effect on NF‐κB expression in fibroblasts stimulated by bacterial lipopolysaccharide and other inflammatory cytokines, including MCP‐1, IL‐8, and IL‐1.

However, some studies have shown an increment in the production of anti‐inflammatory genes simultaneously with an increase in the expression of inflammatory cytokines, indicating the body's attempt to balance the disturbed inflammatory and anti‐inflammatory factors (Barrie et al., 2017). In our study, the lower expression of CB2 receptors in the periodontitis group may be due to the reduced protective effect of anti‐inflammatory agents. These include cannabinoids and the imbalance leading to the predominance of pro‐inflammatory effects. The interaction between anti‐inflammatory and pro‐inflammatory agents is not yet fully understood, so the role of an anti‐inflammatory agent in studies is not possible without considering its interaction with other factors affecting immunity.

As the EC system modulates inflammatory reactions, cannabinoid receptors can be targeted in periodontitis.

There is a shred of considerable evidence for the therapeutic impact of cannabinoids on inflammatory diseases. Thus, the major role of the immune and inflammatory reactions in periodontal disease brings this idea that cannabinoids may benefit the treatment of periodontitis, similar to using cannabinoids mouth spray in the treatment of MS. As the understanding of oral diseases during recent decades improved, immune therapies as a complement to mechanical plaque control methods in the treatment of periodontitis have been considered. Environmental inflammatory factors such as smoking and poor oral hygiene lead to oral diseases that may have destructive effects on the balance of the nervous system and the body's defenses; not only do they have local side effects, but they endanger one's health and well‐being.

In conclusion, as CB2 receptors are expressed in gingival tissues, particularly immune cells and fibroblasts, they involve in tissue and wound repair. The lower expression of these receptors in periodontitis, could be related to the inflammatory reactions and interrupts wound repair. Therefore, it seems that the use of cannabinoid CB2 agonists in the form of mouth wash contributes to the healing of periodontitis.

## AUTHOR CONTRIBUTIONS

*Doing experiments and manuscript drafting*: Atefe Ataei, Amir Moeintaghavi, and Habibollah Ghanbari. *Research director, conception and design of the study, data analysis, reviewing, and finalizing the manuscript*: S.A. Rahim Rezaee and Majid Azizi. All authors have read and approved the final manuscript.

## CONFLICT OF INTEREST

The authors declare no conflict of interest.

### ETHICS STATEMENT

The Research Ethics Committee of Mashhad University of Medical Sciences approved our study by (IR.mums.sd.REC.1394.296). The informed consent was obtained and signed from all subjects for participation in the study.

## Data Availability

Data are available upon reasonable request.
